# Effect of Needle Heating on the Sewing of Medical Textiles

**DOI:** 10.3390/polym13244405

**Published:** 2021-12-15

**Authors:** Adnan Mazari

**Affiliations:** Department of Clothing, Technical University of Liberec, Studenstksa 2 Husova, 46117 Liberec, Czech Republic; adnan.ahmed.mazari@tul.cz

**Keywords:** medical textiles, polyester, sewing, thread

## Abstract

Medical textiles, such as gowns, scrubs, and even disposable uniforms, are all stitched by sewing machines. These garments are mostly made from polypropylene (PP) and polyester due to their durability, antibacterial performance, and functionality. Demand for these garments has significantly risen in the last few years, and sewing machines are able to stitch at extremely high speeds. However, higher sewing speeds can cause burnt spots on the fabric, lower seam strength, and a decrease in production due to thread breakage. In this paper, I have deeply discussed how medical textiles lose their strength and functionality due to higher sewing speeds; this problem is often neglected due to high production demands. This research is based on PP medical gowns, stitched with polyester (PET) threads, sewn at different speeds. The experimental work is also followed by a theoretical explanation of needle heating during the stitching of medical textiles.

## 1. Introduction

Sewing machines are a necessity for any industry working with clothing, automobiles, footwear, medical textiles, or upholstery. Millions of products, ranging from car seat covers to medical gowns to firefighter clothing, are all stitched using industrial sewing machines. Thomas Saint is considered to have invented the practical sewing machine in 1790 [1]. It was a time of industrial revolution, and machines can greatly improve production capacity. The industrial sewing machine, used in the clothing industry, requires not only high production, but also higher sewing quality (seam strength and appearance). Typically, these machines can complete 70–90 stitches in a second [2], to keep the process as efficient as possible. It’s necessary to improve each aspect of the process. Even though sewing machines can run at high speeds, human capability and technical problems during the sewing process forces users to run the machines at the much lower rate at 20–45 stitches per second. There are multiple technical reasons why it is preferable to run machines at less than half capacity, including damage to the thread, burn spots on the fabric, weaker seam strength, and many more [3,4,5]. The factors that cause these problems range from ambient conditions to the process parameters of sewing.

In the modern clothing production sector, there has been significant technological advancement in the last 10 years. One of the key machines used in these production units is the sewing machine, which has been made more robust, efficient, and durable. High production demand makes users run these machine so as to be as productive as possible. As a result of this high-speed production, abrasion within machine components, frictional forces, and penetration forces cause significant impacts on the overall quality of the final seam [6]. The aesthetic, as well as the functional quality, is very important for clothing, especially technical clothing.

Until now, the production of clothing by sewing machine has been labour intensive work, though sooner or later production will move towards automatic sewing. To achieve this, many aspects of the sewing process need to be closely monitored, to understand and improve them for better care-free performance [7,8]. Semi-automatic sewing machines are already on the market, though they still need constant quality management by workers [9]. Often, only thread breakage and aesthetic faults can be controlled [10,11], and all other damage, which may significantly reduce the seam strength, is just avoided. One of these semi-automatic sewing machines is used for jeans pockets. In this research, my focus was on the lockstitch machine due to its versatile usage and application in high strength seams, as compared to the chainstitch mechanism.

In the textile industry, the sewing process is a commonly used method to prepare final clothing. In the last few decades, not only the production, but also the quality, of the sewing has become important. Generally, multiple layers are stitched together using a sewing machine (lockstitch machine), and workers are usually paid according to work done. Therefore, it is very important for workers to produce as many garments as possible, to earn more money. Sewing machines which have the capacity to easily run at 60–100 stitches per second are one of the key solutions for workers aiming to produce and earn more. However, this has its own disadvantages. The sewing needle can reach a temperature of ∼150–320 °C [11]. The hot needle damages the sewing thread and leaves undesirable spots on the fabric. The thread usually loses 35–45% of its strength [12,13].

### 1.1. Medical Gowns

Different varieties of medical apparel are available on the market. Most of them are either stitched by sewing machine or bonded by ultrasonic machine. Generally, all reusable medical gowns and protective gear are stitched with sewing machines for better durability. The material used for making these garments is multiple layers of non-woven spun-bonded PP and PET with functional membranes. In accordance with industry standards, the stitch line is covered with protective tape to avoid leakage of spills or chemicals onto the body. The common standards for medical textiles are EN ISO 13982, EN ISO 13688, EN 13034, EN 14126, and EN 14605.

The effect of sewing speed on these medical textiles is ignored. As medical textiles were often single-use in the past, it was not very important; however, due to industrial waste of medical apparel, the majority of the new gowns are reusable, and are stitched with sewing machines. The current research is focused how sewing speed actually damages the seam strength of medical textiles.

### 1.2. Basic Thermal Mechanism of Needle Heating

The heating of the sewing needle is complicated process; the temperature abruptly rises in the first 10–13 s to above 130 °C [11,12], after which the rise is minor, until a steady state is attained. In each cycle of stitch formation, there are minor variations in the needle temperature during fabric penetration and withdrawal [12].

Heat is produced mainly due to friction between the needle and the thread, as well as between the needle and the fabric. Some researchers have reservations on this subject, and believe that the fabric is a source of cooling, rather than heating, whereas the other group consider it a heat source, due to the penetration and friction with the needle. Researchers from both schools of thought [12,13,14,15,16,17] have published numerous articles on this issue.

- Heat flux is generated between the outer surface of the needle and the fabric; this phenomenon depends on the needle-penetration force, withdrawal, and the frictional coefficient.
- Heat conduction between the needle and the thread is another major source of heating, and depends on the friction coefficient of the needle and thread, as well as the material parameters.

**Figure 1:**
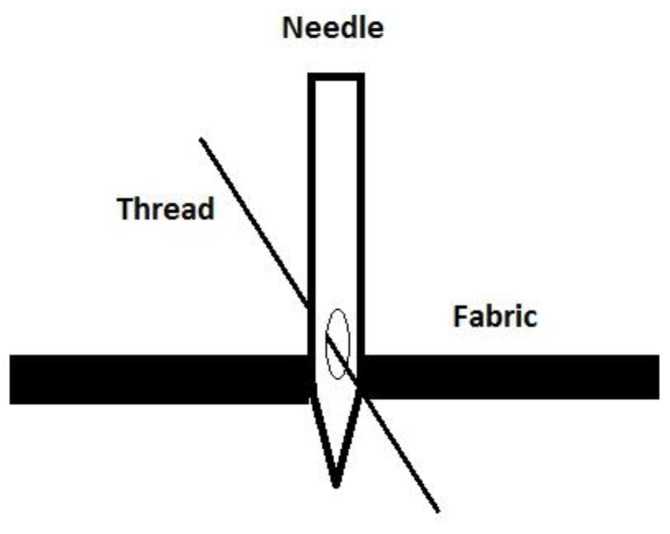
Sewing needle, thread, and fabric diagram [18].

Figure 1 shows the configuration of the sewing process that is responsible for the heating mechanism [18].

### 1.3. Contactless Method of Needle Temperature Measurement

The temperature measurement of stationary objects with high emissivity levels, such as clothing, walls, and paints, is comparatively easy, while in the case of sewing needles, with small size (0.1 to 0.2 cm), low emissivity (0.05–0.1) [19], and a high temperature range (100–300 °C) [18], it is almost impossible to obtain accurate results. In addition, the textile material and sewing thread are too close to the needle, with huge difference in emissivity, making it much harder to focus on only the sewing needle. In the past 10 years, the majority of researchers [20] used the following technique to measure the needle temperature during sewing process.

### 1.4. Sewing Needle Temperature

The temperature measurement of sewing needles is quite complicated due to their small dimension, low emissivity, and high speed during use. There are many methods and techniques used by researchers [4,5,6,7,8,9] to observe needle temperature, including infrared pyrometer, contact sensors, and sensitive waxes/colors, etc. Moreover, with improved knowledge of sewing needle heating, there are many improvements [21,22] to be made in the design of needle, as well as the use of lubricants and coatings, which can help to decrease needle temperature.

## 2. Materials and Methods

For this research, the most common medical-textile material and sewing thread was used to determine the effect of sewing processes on seam strength and needle temperature.

The following fabric as described in the Table 1. was used for the experiment.

The selection of this material was mainly due to its common usage in the production of medical gowns; as well, this material is preferred for reusable gowns in the medical field. The thread which was used in the sewing of these gowns is listed below in Table 2.

Three layers of fabric were stitched together, for 30 s each time, at different sewing speeds, and properties such as tensile strength of the thread, needle temperature, and aesthetic damage to the textile were recorded before and after the sewing process. The stitch length, needle, and ambient conditions were kept constant for all experiments.

Conditions for all experiments were kept constant at 26 °C and 65% RH. The following machine and accessories were used for the experiment.

- Lockstitch machine (Brother Company, Berlin, Germany DD7100-905).
- Thermocouple by Omega (K type 5SC-TT-(K)-36-(36)).
- Thermocouple by Omega—wireless device and receiver (MWTC-D-K-868).
- Needles (Groz–Beckert, Stuttgart, Germany 100/16) R-type.

Initially, the medical-textile material was stitched at different sewing speeds, and the needle temperature was recorded. The methodology used to measure needle temperature was the inserted thermocouple technique, in which a thermocouple is embedded in the needle to obtain an accurate needle temperature; this technique is explained by the authors of [23].

**Figure 2:**
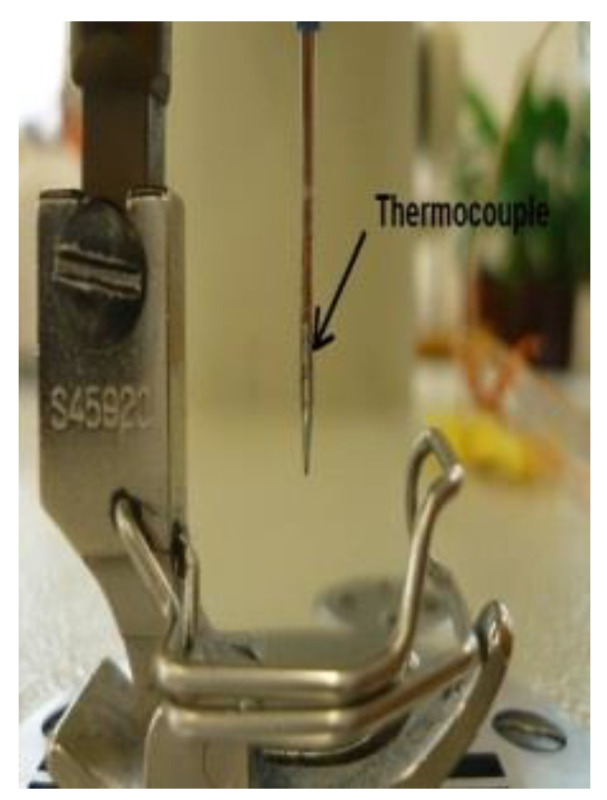
Thermocouple embedded in the needle.

In this method as shown in Figure 2. for measuring sewing needle temperature, a thermocouple, by Omega (K type 5SC-TT-(K)-36-(36)), was inserted into the groove of the sewing needle and soldered. The thermocouple was located near the eye of the needle, to measure the exact needle temperature, and the temperature was measured at different sewing speeds. This method proved to be very efficient, as it provided continuous changes in needle temperature every second, and it had a low standard deviation.

Subsequently, to measure the strength of the threads, the sewing thread was taken out of the seam and the tensile strength of the final thread, after stitching, was compared with its parent strength. The thread was pulled out of the seam by carefully cutting the bobbin thread, without disturbing the twist.

## 3. Results and Discussion

**Figure 3:**
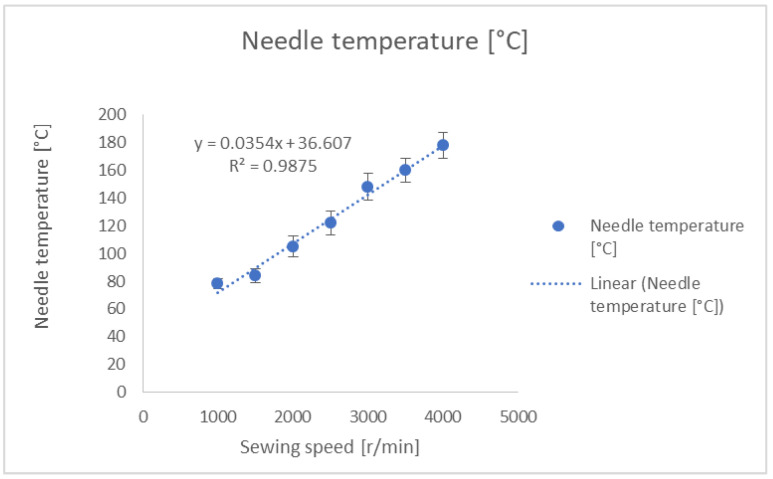
Sewing needle temperature at different sewing speeds.

A continuous 30 s of sewing was performed, keeping all conditions similar for the duration of the experiment. The results of the needle temperature measurement are shown in Figure 3.

At a sewing speed of 3000 r/min, the fabric started to show black spots due to the high temperature of the needle. It can be also seen from the Figure 3 that there was a linear increase in sewing needle temperature with respect to machine speed. Polyester thread has a higher melting temperature than PP, and therefore may not melt; nonetheless, this high temperature causes structural changes to the polymer. 

**Figure 4:**
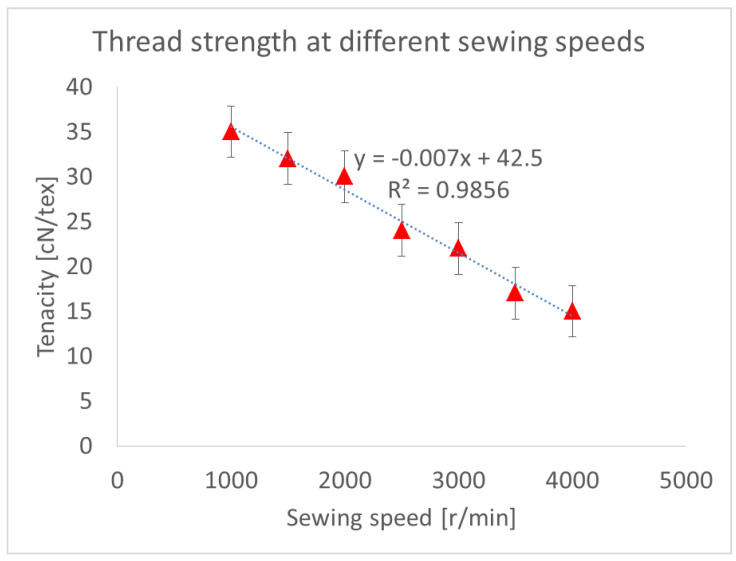
Tensile strength of thread at different sewing speeds.

The tensile strength of the sewing thread was measured using ASTM 2256 standards. The Figure 4 shows the obtained results.

It can be clearly seen that, at higher sewing machine speeds, there was a significant decrease in thread strength, which may have been due to abrasion, or the high temperature of the needle. There was negative linear relationship between sewing speed and thread strength. It was also alarming that, at speeds of 3000 r/min and higher, the thread lost 50% of its original strength.

Figure 3 and Figure 4 show that, with higher speeds, there is a rise in needle temperature and a decrease in the tensile strength of the thread. The highest temperature, 187 °C, was recorded at a speed of 4000 r/min, during which the sewing thread lost more than 50% of its original strength. To explain this needle heating for sewing thread a theoretical model was developed, which is improved work of the author himself.

### Theoretical Analysis

Different methods and approach have been used by researchers in the past, and a major conflict is whether or not the fabric is the source of heating; different schools of thought exist. In my approach, the analytical model is considered, which requires less computation, has higher accuracy, and could be easily used by industrial partners in the future. In this model the assumptions listed below are made:

1- The needle, thread, and fabric are all at room temperature.
2- The needle is a cylinder with the same material composition throughout.
3- *λ_N_* is the thermal conductivity of the needle material, and is significantly higher than that of the sewing thread *λ_y_* and fabric *λ_F_*. Here it is discreetly assumed that both the thread and fabric have lumped thermal properties, i.e., each has unchanging thermal conductivities, represented by single value.
4- The thread and textile fabric are considered homogenous, with constant thermal conductivity value throughout.
5- Radiation heat can be neglected due to the small size of the needle and a smaller contribution compared to other factors.
6- In this model, it is estimated that friction heat is assumed as Q = F*v [18], where F is friction force and v is the relative velocity of the two surfaces.
7- *γ* is the ratio determining the amount of heat distribution when two materials rub together. Partition ratio is calculated, using the Charron’s relation [24], as:
  (1) $$\gamma =\frac{1}{1+\xi_{N}}$$
  where $\xi_{N}=\frac{b_{i}}{b_{N}}$, *N* denotes the needle, *i* denotes the other rubbing material in contact, and *b* is the thermal absorptivity of the respective materials. The calculated value is given as $b=\sqrt{\left(\rho *C*\lambda \right)}$, where *ρ* is the density of the material, *C* is the specific heat of the material, and λ is the thermal conductivity.

An analytical approach was used to predict needle temperature, assuming that heat is produced during the sewing process as a result of needle–thread and needle–fabric friction. In this analysis, a steady-state condition is considered, in which the amount of heat generated by friction exactly equals the amount of heat lost by the needle. The complex shape of needle is neglected, and it is treated as a uniform cylinder.

The heat generated due to friction between the needle and the fabric can be expressed as [18]:

(2) $$Q_{FN}=\gamma_{FN}*\mu_{FN}*F_{FN}*v_{FN}$$

The heat generated due to friction between the sewing thread and the surface of needle can be expressed as:

(3) $$Q_{YN}=\gamma_{YN}*\mu_{YN}*T_{y}*\cos\theta *v_{YN}$$

where the legends are described in Table 3.

Maximum needle speed was found to have a linear function of machine speed with the multiplier constant *C_FN_* = 0.0008.

The total heat gain by the needle is finally:

(4) $$Q_{N}=Q_{FN}+Q_{YN}$$

From the 1st law of thermodynamics in a closed system,

(5) $$Q=m*C_{N}*\left(T-T_{i}\right)$$

where *m* is the mass of the needle, *C_N_* is the specific heat of needle, *T* is the final temperature of needle, and *T_i_* is the initial temperature of needle

From Equations (1)–(5), following relation is obtained:

(6) $$m*c_{N}*\left(T-T_{N}\right)=\gamma_{FN}*\mu_{FN}*F_{FN}*v_{FN}+\gamma_{YN}*\mu_{YN}*T_{y}*\cos\theta *v_{YN}$$

Equation (6), for a more precise result, should be solved by calculating it numerically over time, as many of the variables present in Equation (6) are complex functions of time. However, in order to simplify the calculations, the maximum value of *F_FN_* and *T* are considered here for the prediction of maximum needle temperature. Similarly, the maximum relative speed between the needle and sewing thread is used as $v_{YN}$. As an additional approximation, both $v_{FN}$ and $v_{YN\;}$are stated as proportional to the machine speed $v_{M}$. If *C_FN_* and *C_YN_* are the two coefficients of these proportionalities, respectively, then it can be obtained from Equation (6) that:

(7) $$T-T_{i}=B*v_{M}$$

where “*B*” is:

(8) $$B=\frac{1}{m\times c_{N}}*\left\{\gamma_{FN}*\mu_{FN}*F_{FN}*C_{FN}+\gamma_{YN}*\mu_{YN}*T*\cos\theta *C_{YN}\right\}$$

Thus, Equation (8) shows that the highest needle temperature is a linear function of machine speed. An estimate of maximum needle temperature, from machine speed, is possible if the parameter *B* can be calculated using Equation (8). The value of the symbols are described in Table 4.

**Figure 5:**
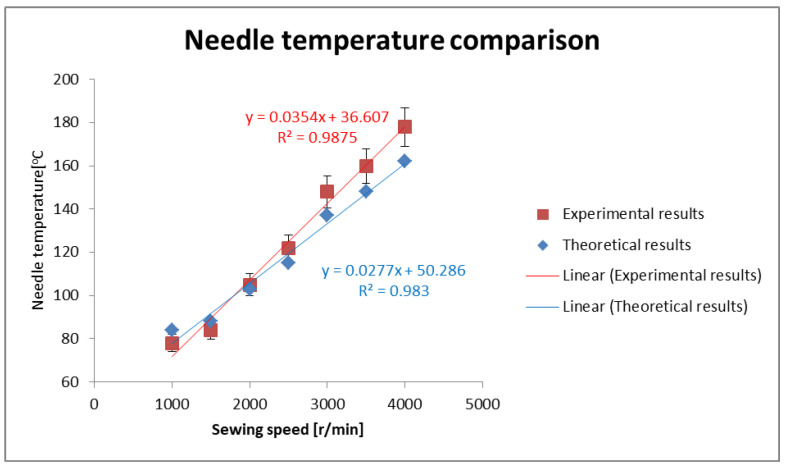
Comparison of experimental and theoretical results.

It can be seen from Figure 5 that the theoretical and experimental results both show a linear trend of increasing needle temperature. A difference in absolute values is possible, as in all theoretical models many assumptions are made to simplify the model. The analytical approach can be solved without complex simulation.

## 4. Conclusions

The process of sewing is very important for any clothing or wearable textile company. Any improvement in the process can provide huge economic benefits, including higher productivity. Generally, the strength of the seam, or sewing thread, used in the stitching of medical textiles is neglected. As the demand for reusable medical textiles, such as gowns, is increasing, it is important to understand the performance parameters of the seams and sewing threads used in the stitching of medical textiles. The focus of this research was the theoretical and experimental explanation of how needle heating due to machine speed can impact the strength of medical textiles. This work provides in-depth knowledge of the sewing process, including possible limitations and improvements to be made in the textile industry. This work is also beneficial for researchers, as it provides a theoretical analysis of sewing needle temperature measurement. If cooling accessories are not used, it is advisable to run the sewing machine at a speed of 2000 r/min or lower.

## Figures and Tables

**Table 1:** Fabric details.

Material	Manufacturing Technique	Weight [g/m^2^]	Thickness [mm] (S.D)	Layers Used
PP	Spun-bonded melt-blown polypropylene	75	1.2 (±0.15)	3

**Table 2:** Thread details.

Material	Twist	Count [tex]	Producer
PET	Z/S	40	Amman^®^ (Liberec, Czech Republic)

**Table 3:** Legend of equations.

$\gamma_{NY}$	Partition ratio of heat gain between needle and thread using Charron’s relation.
$\gamma_{FN}$	Partition ratio of heat gain between needle and fabric using Charron’s relation.
$\mu_{YN}$	Coefficient of friction between needle and sewing thread.
$\mu_{FN}$	Coefficient of friction between fabric and sewing thread.
$T_{y}$	Maximum tension of sewing thread during sewing cycle.
θ	Angle of sewing thread with respect to the needle.
$v_{FN}$	Velocity of needle with respect to the fabric.
$F_{FN}$	Needle penetration force with respect to the fabric.
$v_{YN}$	Velocity of thread with respect to the needle.

**Table 4:** List of symbol and units.

Property	Symbol	Value	Unit
Heat partition ratio (fabric & needle) [24].	$\gamma_{NF}$	0.945	-
Heat partition ratio (thread & needle) [24].	$\gamma_{NY}$	0.958	-
Density thread [25].	ρ *_y_*	1400	kg/m^3^
Specific heat of thread [26].	*C_y_*	750	J/kgK
Thermal conductivity of thread [26].	λ *_y_*	0.15	W/mK
Density of fabric [27].	ρ *_f_*	920	kg/m^3^
Specific heat of fabric [26].	*C_f_*	1700	J/kgK
Thermal conductivity of fabric [experimental].	λ *_f_*	0.04	W/mK
Density of needle [28].	ρ *_n_*	7850	kg/m^3^
Specific heat of needle [28].	*C_n_*	523	J/kgK
Thermal conductivity of needle [28].	λ *_n_*	40	W/mK
Friction coefficient of needle and thread [experimental value].	$\mu_{t}$	0.3	-
Friction coefficient of needle and fabric [experimental value].	$\mu_{fy}$	0.21	-
Tension thread maximum [29,30].	$T_{y}$	1.05	N
Needle velocity [experimental value].	$v_{N}$	2.3	m/s
Machine speed (to be used with constant to balance the equation).	*V_m_*	1000–4500	r/min
Needle and thread angle of contact [31].	θ	60	^o^
Frictional normal penetration force to needle from fabric [experimental value].	$F_{FN}$	2.5	N

## Data Availability

Not applicable.
